# Is There an Interplay between Environmental Factors, Microbiota Imbalance, and Cancer Chemotherapy-Associated Intestinal Mucositis?

**DOI:** 10.3390/ph17081020

**Published:** 2024-08-03

**Authors:** Camila Fernandes, Mahara Coelho Crisostomo Miranda, Cássia Rodrigues Roque, Ana Lizeth Padilla Paguada, Carlos Adrian Rodrigues Mota, Katharine Gurgel Dias Florêncio, Anamaria Falcão Pereira, Deysi Viviana Tenazoa Wong, Reinaldo Barreto Oriá, Roberto César Pereira Lima-Júnior

**Affiliations:** 1 Department of Physiology and Pharmacology, and Drug Research and Development Center (NPDM), Faculty of Medicine, Federal University of Ceara, Rua Cel Nunes de Melo, 1000, Fortaleza 60430-270, Brazil; camilafarmaco@gmail.com (C.F.); analizeth_pp@yahoo.es (A.L.P.P.); adrianm@aluno.ufc.br (C.A.R.M.); katharineflorencio@gmail.com (K.G.D.F.); anamariafp0507@gmail.com (A.F.P.); deysiviviana@ufc.br (D.V.T.W.); 2 Graduate Program in Oncology, Haroldo Juaçaba Hospital, Cancer Institute of Ceara, Fortaleza 60430-230, Brazil; maharacoelho@gmail.com; 3 Laboratory of Tissue Healing, Ontogeny, and Nutrition, Department of Morphology, and Institute of Biomedicine, Faculty of Medicine, Federal University of Ceara, Fortaleza 60430-170, Brazil; roquecassia@yahoo.com.br (C.R.R.); oria@ufc.br (R.B.O.)

**Keywords:** cancer, chemotherapy, intestine, xenobiotics, mucositis, diarrhea

## Abstract

Interindividual variation in drug efficacy and toxicity is a significant problem, potentially leading to adverse clinical and economic public health outcomes. While pharmacogenetics and pharmacogenomics have long been considered the primary causes of such heterogeneous responses, pharmacomicrobiomics has recently gained attention. The microbiome, a community of microorganisms living in or on the human body, is a critical determinant of drug response and toxicity. Factors such as diet, lifestyle, exposure to xenobiotics, antibiotics use, illness, and genetics can influence the composition of the microbiota. Changes in the intestinal microbiota are particularly influential in drug responsiveness, especially in cancer chemotherapy. The microbiota can modulate an individual’s response to a drug, affecting its bioavailability, clinical effect, and toxicity, affecting treatment outcomes and patient quality of life. For instance, the microbiota can convert drugs into active or toxic metabolites, influencing their efficacy and side effects. Alternatively, chemotherapy can also alter the microbiota, creating a bidirectional interplay. Probiotics have shown promise in modulating the microbiome and ameliorating chemotherapy side effects, highlighting the potential for microbiota-targeted interventions in improving cancer treatment outcomes. This opinion paper addresses how environmental factors and chemotherapy-induced dysbiosis impact cancer chemotherapy gastrointestinal toxicity.

## 1. Introduction

Interindividual variation in drug response, in terms of efficacy and toxicity, is a significant problem that leads to adverse clinical and economic public health outcomes. For a long time, pharmacogenetics and pharmacogenomics have been claimed to cause such heterogeneous responses [1]. Recently, pharmacomicrobiomics has gained attention as a possible explanation for the variability in drug response and toxicity. It mainly highlights the microbiome as a critical determinant [2].

The microbiome is a community of microorganisms (such as bacteria, fungi, protozoa, and viruses) and their genomes living in or on the human body [3]. Diet, lifestyle, exposure to xenobiotics, use of antibiotics, acute and chronic illness [4], geographic location, ethnicity, age, physical activity, genetics, and gender can influence the composition of the microbiota [5]. For example, a high-fat diet has been shown to alter the gut microbiota composition, leading to a higher abundance of bile-tolerant microorganisms [6]. Similarly, antibiotic use can significantly reduce microbial diversity, affecting drug metabolism and response [7].

Variations in the intestinal microbiota, a vital part of the human microbiome, and their relationship with drug responsiveness have been extensively investigated [8,9,10,11,12]. Such interaction follows a bidirectional and complex interplay. Pharmacological treatment can influence the composition of the microbiota [3,13,14], but the microbiota can also modulate an individual’s response to a drug, influencing its bioavailability, clinical effect, and toxicity [3,14]. For instance, age-related microbiome changes can affect drug pharmacokinetics, as seen in the reduced efficacy of certain medications in older adults [15]. Gender differences have also been observed, with studies indicating that intestinal microbiota composition drives hormone metabolism and the regulation of autoimmunity [16]. Lifestyle factors, including voluntary wheel running and forced treadmill running, were reproduced in rodents and have been demonstrated to alter the intestinal microbiome of mice differentially [17]. Additionally, xenobiotics, including anticancer agents, can disrupt the microbiome, leading to increased gastrointestinal toxicity [18].

**Figure 1:**
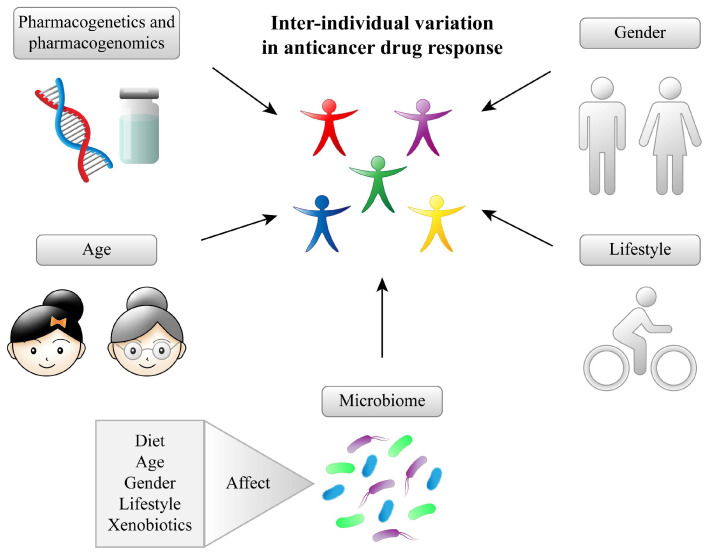
Factors that influence the intestinal microbiome balance. Interindividual variations in cancer treatment response can be influenced by factors such as pharmacogenetics and pharmacogenomics, gender, age, lifestyle, and changes in the microbiome. The microbiome can be influenced by diet, age, sex, lifestyle, xenobiotics (including anticancer agents), low socioeconomic status, and poor sanitation.

Figure 1 summarizes some factors that influence the intestine microbiome balance.

Cancer chemotherapy presents a narrow therapeutic index and a high rate of individual variation in treatment response. Remarkably, individual alteration in the level of toxicity has recurrently been described [19]. Cancer chemotherapy side effects hamper cancer treatment outcomes. Patients frequently manifest several gastrointestinal symptoms, such as nausea, vomiting, mucositis, and diarrhea [20]. Intestinal mucositis is a dose-limiting side effect of chemotherapy occurring in about 50–80% of patients, negatively affecting their treatment and prognosis [21]. Changes in intestinal microbiota composition are related to interindividual variations in patients undergoing intravenous chemotherapy with 5-fluorouracil, cyclophosphamide, irinotecan, oxaliplatin, gemcitabine, and methotrexate. Probiotics modulate the microbiome and ameliorate chemotherapy side effects in animal models and humans [21,22,23,24,25,26]. Herein, we address the relationship between changes in the microbiome from any causes and how they impact cancer chemotherapy and gastrointestinal toxicity development.

## 2. Environmental Factors Alter Intestine Microbiome Composition

**Figure 2:**
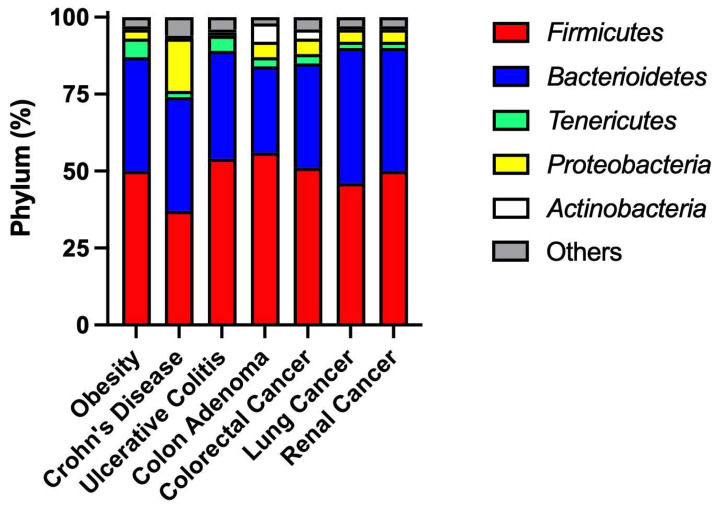
Impact of common clinical conditions on the composition of the most predominant gut microbial phyla. Data were adapted from public Human Gut Microbiome Atlas databases, available at https://www.microbiomeatlas.org (accessed on 28 May 2024). In this figure, the microbial data were obtained at the phylum level from a total sample size of 1165 samples, comprising 164 samples from subjects with obesity, 71 from subjects with Crohn’s disease, 21 from subjects with ulcerative colitis, 156 from subjects with colon adenoma, 534 from subjects with colorectal cancer, 118 from subjects with non-small cell lung cancer, and 101 from subjects with renal cancers. The corresponding published articles ensure that the necessary ethical approvals regarding data access were obtained. The primary publications included the cohorts from the following bioprojects: Obesity (PRJEB4336); Crohn’s disease (PRJEB15371; PRJEB2054); Ulcerative colitis (PRJEB2054); Colon adenoma (PRJDB4176; PRJEB6070; PRJEB7774; PRJNA447983); Colorectal cancer (PRJDB4176; PRJEB10878; PRJEB12449; PRJEB27928; PRJEB6070; PRJEB7774; PRJNA447983; PRJNA531273); Non-small cell lung cancer (PRJEB22863); Renal Cancer (PRJEB22863).

The intestinal microbiota consists of more than 1500 species. The phyla *Bacteroidetes* and *Firmicutes* are the most predominant and, together with *Proteobacteria*, *Fusobacteria*, *Tenericutes*, *Actinobacteria*, and *Verrucomicrobia*, represent around 90% of the human microbial population [27,28]. Figure 2 shows that microbiome variability in the gut is observable among disease conditions, such as obesity, Crohn’s disease, ulcerative colitis, colon adenoma, and cancers of the colon and rectum, lung, and kidney. No phyla are universally present among disease types, and every individual is characterized by their signature, indicating the plurality of the microbial community, suggesting the relevance of environmental factors. Additionally, diet changes and drug exposure account for over 20% of interindividual microbiome variability [29].

### 2.1. Diet

Dysbiosis is defined as changes in the function and composition of the intestinal microbiota, which may be implicated in the pathogenesis of several diseases, including cancer and inflammatory diseases [30]. Diet, host genetics, infection, and inflammation are critical players that trigger the transition from healthy to dysbiosis states [30]. Among the dietary metabolites, a high-fat diet [31], as well as sugar and processed foods [32], significantly drive intestine dysbiosis. Epidemiological evidence indicates that individuals with plant food- and fiber-based diets have greater microbial diversity and a predominance of *Prevotella* over *Bacteroides*. By contrast, those with a diet rich in fat, sugar, and processed foods have significant quantities of *Proteobacteria* [32].

An exploratory randomized controlled trial compared the effects of microcrystalline cellulose, which is not fermented by the gut microbiota, and high supplementation doses of arabinoxylan, a long-chain complex dietary fiber, on gut microbiota composition and short-chain fatty acid production. The study was conducted over six weeks in individuals with overweight and class-I obesity, and it was concluded that arabinoxylan supplementation globally improves fecal bacterial community composition, promotes specific taxa, including *Bifidobacterium longum*, *Blautia obeum*, and *Prevotella copri*, and increases the production of propionate, a short-chain fatty acid (SCFA) [33].

Some strains of *Prevotella* are associated with more variable SCFA production from the same substrate than *Bacteroides* [34]. SCFAs are critical factors in regulating the intestinal epithelial barrier and anti-inflammatory properties of the rich-fiber diet [35]. Remarkably, raising colonic propionate delivery in humans modifies gut bacterial diversity and significantly lowers markers of systemic inflammation, such as interleukin-8 [36], a neutrophil chemoattractant cytokine. Additionally, SCFAs were shown to regulate intestinal adaptive immune responses and promote health in a murine model of colitis by raising immunosuppressive Treg cell levels [37]. It contrasts with the pro-inflammatory effects of long-chain fatty acids that enhance the differentiation and proliferation of T helper 1 (Th1) or Th17 cells in the small intestine [38].

The *Proteobacteria* phylum is numerically reduced in a healthy intestine, whereas the increase in the gamma-*Proteobacteria* class and the *Enterobacteriaceae* family are associated with human inflammatory diseases [39]. Gut dysbiosis in a dextran-sodium sulfate rodent model of colitis is knowingly associated with loss of intestinal barrier function, allowing for the translocation of microbial products across the intestinal epithelium where they induce an inflammatory response [40]. Notably, a fiber-rich diet regulates tight junction barrier integrity and intestinal homeostasis, attenuating intestinal inflammation by elevating SCFA production [41]. The mechanism might involve the overgrowth of enteropathogenic bacteria [39].

Therefore, the emergence of pathogenic microbes from an unbalanced low-fiber diet facilitates the inflammatory response triggered by other insults.

### 2.2. Xenobiotics

Xenobiotics, such as heavy metals, antibiotics, cancer chemotherapy drugs, pesticides, artificial sweeteners, and others, can impact the composition of the intestine microbiota and, consequently, local homeostasis, ultimately affecting the individual’s health.

Antibiotics adversely influence the intestinal microbiota, ranging from reduced species diversity and alteration in metabolic activity to the selection of resistant microorganisms. Orally administered vancomycin reduces intestinal microbiota diversity, which affects bile acid metabolism and reduces peripheral insulin sensitivity [42]. The administration of a cocktail of antibiotics, gentamicin, meropenem, and vancomycin, causes pathobionts to predominance and reduce the number of *Bifidobacteria* species [43]. Antibiotic treatment may also favor intestine colonization by toxigenic strains of *Clostridioides difficile* and the development of diarrhea in susceptible patients [44]. Eliminating bacterial populations in the gastrointestinal tract by antibiotic exposure reduces the secretion of antimicrobial peptides, an essential mechanism for controlling pathogenic bacteria growth [45]. Additionally, antibiotics diminish ZO-1, occludin, and claudin expression, enhancing intestinal permeability and microvilli disruption [46,47]. Antibiotic therapy to treat or prevent infections can also contribute to dysbiosis [48].

Among the xenobiotic environmental pollutants, heavy metals such as arsenic and cadmium are related to the development of dysbiosis. They can reduce the relative abundance of *Firmicutes* and the production of SCFAs [49,50]. Mercury is another heavy metal whose oral exposure also causes intestinal dysbiosis in mice [51,52]. Pesticide contamination is an additional public health problem related to intestine microbiota changes. It is particularly worrisome due to the wide use of pesticides and possibly excessive amounts in food and groundwater reservoirs. Contaminated food or drinking water first passes the gastrointestinal tract, the primary barrier affected by pesticide effects in the body. Additionally, in vitro studies indicate that exposure of intestinal bacteria to the pesticide Glyphosate dramatically decreases their proliferation [53]. Organophosphate pesticides significantly reduce commensal bacteria, such as *Lactobacillus* and *Bifidobacterium*, and promote *Enterococcus* and *Bacteroides* overgrowth [54]. Notably, permethrin, one of the most representative pyrethroid pesticides for residential use, shows higher antibacterial activity against beneficial bacteria, including *Bifidobacterium* and *Lactobacillus paracasei* [55].

Several studies evaluated the impact of anticancer chemotherapy on the intestinal microbiota [19]. Patients with non-Hodgkin’s lymphoma treated with high doses of etoposide (a topoisomerase II inhibitor) and alkylating agents carmustine (bis-chloroethyl nitrosourea) and melphalan present with microbiota changes one week after the start of chemotherapy. There was a drastic reduction in fecal bacterial DNA expression of *Faecalibacterium* and an increase in *Escherichia* [56]. Remarkably, Motoori and colleagues found a reduced population of *Lactobacillus* in patients with esophageal cancer undergoing 5-fluorouracil (an antimetabolic drug), cisplatin (an alkylating agent), and docetaxel (a taxane with anti-mitotic action)-combined chemotherapy treatment, while the population of *Clostridoides difficile* and *Enterococcus* increased significantly [57]. Changes in the intestine microbiota are also described in children with acute lymphoblastic leukemia after receiving high doses of the antimetabolite methotrexate chemotherapy, with a 29.6% reduction in bacterial load, especially in *Bifidobacteria*, *Lactobacillus*, and *E. coli* species [58]. Notably, the decrease in beneficial bacteria can last up to a year after chemotherapy [59].

In rats, intravenous chemotherapy with irinotecan increases the shedding of Clostridium cluster XI and *Enterobacteriaceae* in feces [60]. Irinotecan is a topoisomerase I inhibitor used for treating colorectal and gastric cancers. In models in which irinotecan was administered by intraperitoneal route, it increased the number of β-glucuronidase-producing bacteria, augmenting the production of the active metabolite SN-38 and intestinal mucositis-related bacteria [61]. Chemotherapy-born inflammation resulting from cellular injury can influence intestinal microbiota composition, facilitating the overgrowth of highly metabolic bacteria capable of capturing host nutrients, such as those of the *Enterobacteriaceae* family [62]. D-methionine can prevent intraperitoneal cisplatin-induced toxicity by rising pro-homeostatic bacteria (*Lachnospiraceae* and *Lactobacillus*), facilitating its known antioxidant and anti-inflammatory effects, which improves cisplatin-induced microbiome imbalance [63]. Remarkably, anticancer chemotherapeutic drugs are seldom administered orally; they are primarily delivered intravenously. Notably, the above-referenced studies employed intravenous and intraperitoneal routes for drug administration. Drug enterohepatic circulation may be the primary cause of microbiome alterations [64].

The impact of anticancer drugs as xenobiotics on the intestinal microbiota may extend beyond inducing side effects in the gastrointestinal tract. Microbiome alterations can also have clinical implications for various cancers, including breast, colorectal, lung, prostate, and stomach cancer [65].

Colorectal cancer is the most studied cancer type demonstrating a clear association with gut microbiota dysbiosis, as lifestyle and dietary factors uniquely alter the local microbiota [65]. The mechanisms involve inflammation and the production of carcinogenic products, amplifying DNA damage in intestinal cells [66]. This impact is not restricted to local effects. Understanding the involvement of dysbiosis in the development and progression of other cancers is evolving. A recent meta-analysis investigated breast cancer patients’ fecal, tumor, or oral microbiome profile, suggesting that differences in microbiota abundance by menopausal status, menarche, and cancer stages and changes in the microbial pattern might occur after chemotherapy, impacting patients’ quality of life.

These modifications in the microbiota composition caused by environmental contaminants/stressors can affect intestinal permeability, digestion, metabolism, and immune responses. Eventually, such disturbances can lead to intestine distress, amplifying local damage and diarrhea triggered by anticancer chemotherapy exposure.

## 3. Intestinal Mucositis: Definition and Pathophysiology

Intestinal mucositis is inflammatory-driven damage to the intestinal mucosa that manifests as a side effect of anticancer chemotherapy and is marked by structural, functional, and immunological changes in the mucous membrane lining [67,68]. Intestinal mucositis often contributes to chemotherapy regimen failure and is manifested in a dose-dependent manner [69]. Additionally, such a condition reduces patients’ quality of life and increases healthcare costs, hospitalization, and death [67,69]. Signs and symptoms include nausea, vomiting, pain, ulceration, bleeding, constipation, and diarrhea [70]. These symptoms increase the risk of local and systemic infections and sepsis, delaying subsequent chemotherapy cycles, dose reductions, and treatment discontinuation [67]. The anticancer drugs most involved with this side effect development are irinotecan, 5-FU, taxanes, and capecitabine. In clinical trials, one out of five patients exposed to irinotecan develop severe mucositis. Notably, diarrhea is the most common symptom [71].

Mucositis mechanisms are described based on a five-phase pathophysiology model [68]. It includes 1—initiation; 2—upregulation and message; 3—signaling and amplification; 4—ulceration and inflammation; 5—tissue healing. Briefly, phase 1 consists of tissue damage initiation with the marked release of reactive oxygen species (ROS). Phases 2 and 3 involve the activation of nuclear transcription factor kappa B (NF-kB), leading to massive pro-inflammatory cytokine gene and protein expression, further amplifying NF-kB inflammatory signaling. In phase 4, mucosa ulceration (intestinal epithelial barrier impairment), dysbiosis, intestine-to-blood bacterial translocation, and overproduction of inflammatory cytokines amplify tissue damage-related signaling cascades. Finally, the healing phase occurs due to rapid epithelial renewal under the intestinal stem cell niche modulation with the restoration of the homeostatic microbiota. In each mucositis phase, the microbiota, abundant in the intestine, seems to play a pivotal role by affecting the mechanisms of inflammation, oxidative stress, intestinal permeability, mucus layer composition, epithelial repair, and the immune response [68,72,73,74].

In irinotecan-induced intestinal mucositis, the primary initiation insult depends on the release of the irinotecan-active metabolite SN-38. It causes apoptosis of crypt epithelial cells, hypoplasia of the crypt with epithelial tight junction impairment, and increased bacterial translocation to the intestinal lamina propria [67]. Cell injury releases damage-associated molecular patterns (DAMPS) and pathogen-associated molecular patterns (PAMPS). They are recognized by pattern recognition receptors, such as Toll-like receptors (TLRs), on innate immune and epithelial cells [67,72,75]. Recognition of DAMPS and PAMPS activates immune resident cells and pro-inflammatory cytokine release, accompanied by the mucosal influx of neutrophils [67,72,76,77,78]. Interestingly, irinotecan hampers TLR4 signaling. It affects the recognition of translocating bacteria from the intestine during the chemotherapy-derived acute insult [79]. Such a mechanism amplifies a harmful TLR9-dependent late inflammatory response [73]. Remarkably, *Tlr4*-gene-depleted mice display an altered functional capacity of the intestinal microbiome following irinotecan treatment [80]. How the altered intestinal microbiota drives mucositis development is still controversial. Some clues were found in animal models of inflammatory bowel diseases. Wild-type and *Tlr4* knockout mice show different susceptibilities to intestinal inflammation. Possibly, local microbiota strongly influences disease development. The bacterial profile affects the adaptive immune response, such as RORγt+ Treg cells, modulating the injury [80,81]. In line with those findings, the increased frequency of intestinal Th17 cells and regulatory T cells (Tregs) and an increased Treg/Th17 ratio have been documented in a murine irinotecan intestinal mucositis model as part of the late-onset inflammatory mechanisms. Accordingly, the experimental depletion of Tregs worsens the development of mucositis [82]. These findings reinforce the pivotal immunomodulating role of the microbiota on chemotherapy-associated inflammatory response in the intestine.

## 4. The Interplay between Microbiome and Chemotherapy Toxicity in the Intestine

### 4.1. Immunomodulation

The microbiota is essential for the generation and maintenance of intestinal immune homeostasis. Germ-free mice present with reduced frequency of intestinal Treg cells [83], intraepithelial lymphocytes [84], and compromised innate lymphoid cell function [85]. Self-limited immune and inflammatory responses against pathogens are needed to maintain intestinal health. It can be facilitated by the right balance of commensal bacteria and their metabolites [86] under a healthy human gastrointestinal state of low-grade and time-limited inflammation. The anti-inflammatory properties of members of the microbiota are demonstrated in several studies. *Bacteroides fragilis*, an ordinary member of the microbiota, may stimulate the generation of IL-10-producing Treg cells [87]. Acute/chronic and more severe exposure to intestinal pathogens and their products may disrupt the balanced intestinal environment and burst inflammation and dysbiosis, a typical process during cancer chemotherapy exposure.

Dietary fiber-derived SCFAs generated via bacterial metabolism exert an intestinal anti-inflammatory effect [88], acting through the interaction with G protein-coupled receptors [89] and the histone acetylase pathway [90]. SCFAs regulate TLRs and NLRP3 inflammasome-mediated signaling by inhibiting TLR4 expression and suppressing the histone acetylation pathway [91]. In addition to this, SCFAs reduce intestinal permeability [92]. The activation of NF-kB, a TLR4 downstream signaling transcription factor, is reduced in the presence of *Bacteroides thetaiotaomicron* and *Bifidobacterium infantis* anaerobic commensals [93]. Commensal microbiota can positively influence the composition of the mucus layer lining the epithelium, thus protecting the host against invasive bacteria. Strengthening the epithelial barrier reduces bacterial translocation and inflammation [74]. The reduction in NF-κB activation by a healthy microbiota can also attenuate inflammation through the balance of pro- and anti-inflammatory responses [94,95]. Conversely, chemotherapy-induced dysbiosis can alter TLR signaling pathways, favoring inflammation development [95].

The commensal bacteria stimulate the production of antimicrobial peptides, generating and activating type 3 innate lymphoid cells (ILC3) [96]. ILC3, when activated by TLR2 agonists by commensal bacteria, produces IL-22 [97]. Il-22 stimulates intestinal epithelium renewal, the production of antimicrobial peptides, such as Reg3g, by intestinal epithelial cells, and maintenance of the barrier function by modulating tight junction proteins [98]. Notably, the percentage of ICL3 is reduced in experimental irinotecan-induced intestinal mucositis (unpublished data by our group).

As aforementioned, intestinal dysbiosis is at the front stage of intestinal mucositis development. The onset of chemotherapy-induced severe mucositis coincides with reduced microbial diversity [74]. Conversely, current guidelines support the administration of probiotics for alleviating chemotherapy-related intestinal mucositis symptoms [21,25,26]. The *Lactobacillus casei* variety *rhamnosus* (Lcr35) or *Lactobacillus acidophilus* and *Bifidobacterium bifidum* (LaBi) can improve 5-fluorouracil-induced intestinal mucositis in a mouse model [99]. In another murine model, daily administration of probiotics during 5-fluorouracil treatment improved diarrhea and body weight, restored jejunal crypt depth, and inhibited the production of TNF-α, IL-1β, IFNγ, IL-6, IL-4, and IL-17 [100]. Oral pretreatment with *Bifidobacterium longum* reduced the daily disease activity index, protected the intestinal architecture, preserved the length of the intestine, and reduced intestinal permeability, inflammation, and oxidative damage induced by irinotecan in mice [101]. Accordingly, fecal microbiota transplantation prevents 5-fluorouracil/oxaliplatin-induced toxicity in mice and increases the expression of TLRs, MyD88, and serum IL-6 levels [102]. Antibiotics exacerbate diarrhea induced by chemotherapy with methotrexate, whereas they can be inhibited by autologous fecal microbiota transplantation [103]. Live bacteria and their products can modulate the intestinal immune response with clear benefits on chemotherapy-associated mucositis. Paraprobiotic *Enterococcus faecalis* EC-12 prevents the development of intestinal mucositis by downregulating TLR4 expression and macrophage accumulation [104,105].

A randomized, double-blind, placebo-controlled pilot study corroborates the experimental findings. It showed that colorectal cancer patients undergoing irinotecan-based therapy and treated with probiotics presented reduced incidences of grade 3 or 4 diarrhea and the overall incidence of enterocolitis [106].

### 4.2. Microbiota Effects on the Metabolism of Cancer Chemotherapy Drugs

Intestinal microbiota participates in drug metabolism through enzyme production or microbiota–host co-metabolism. Changes in microbiota are associated with interindividual variation in response to drug therapy [107], and these might influence the response of cancer patients to treatment.

Accordingly, irinotecan is transformed by carboxylesterases into the toxic active form SN-38. SN-38 is then conjugated to glucuronide on SN-38G in the liver before being secreted into the intestine. Eventually, SN-38G can be converted to SN-38 by the β-glucuronidase enzyme of intestinal bacteria, increasing the drug’s toxicity [108].

Dysbiosis with an increased *Escherichia coli* population (and other *Enterobacteriaceae*) in mice with irinotecan-induced mucositis has been associated with the increased production of β-glucuronidase. Such a condition accumulates SN-38 in the intestine and induces drug toxicity [61,109]. However, evidence indicates a partial role of β-glucuronidase in intestinal mucositis induction since pretreatment with antibiotics reduces the content of β-glucuronidase in stools without affecting SN-38 concentrations [67,110]. Interestingly, germ-free mice develop milder intestinal mucositis than specific pathogen-free counterparts, a pattern reversed by germ-free mice conventionalization with normal intestinal microbiota [111]. Furthermore, gnotobiotic mice mono-associated with a β-glucuronidase-producing-*E. coli* strain showed increased intestinal permeability compared to those gnotobiotic mice mono-associated with an *E. coli* strain under β-glucuronidase encoding gene deletion. The role of microbiota bacteria in converting 5-flucytosine (5-FC) to 5-fluorouracil, a cytotoxic chemotherapeutic agent, and increased antifungal 5-FC toxicity has also been described [112].

Intestinal microbiota also modulates the pharmacological effects and toxicities of several other chemotherapeutics, including 5-fluorouracil, cyclophosphamide, oxaliplatin, gemcitabine, and methotrexate, through various mechanisms [113]. The microbiota changes the immune and inflammatory responses triggered by anticancer drugs, such as cyclophosphamide. For instance, *Lactobacillus* mediate the accumulation of type 17 T helper (TH17) cell and type 1 T helper (TH1) cell responses [114]. Additionally, the microbiota fosters indirect metabolic processes, including reduction, hydrolysis, and dealkylation, which alter methotrexate and gemcitabine metabolism. Chemotherapy also reduces mucosal and fecal microbiota, leading to pathobiont predominance, intestinal inflammation, and diarrhea [113]. Therefore, understanding the factors that alter the microbiome allows for a foreseeable impact of pharmacomicrobiomics in cancer patients’ responses to therapeutics.

**Figure 3:**
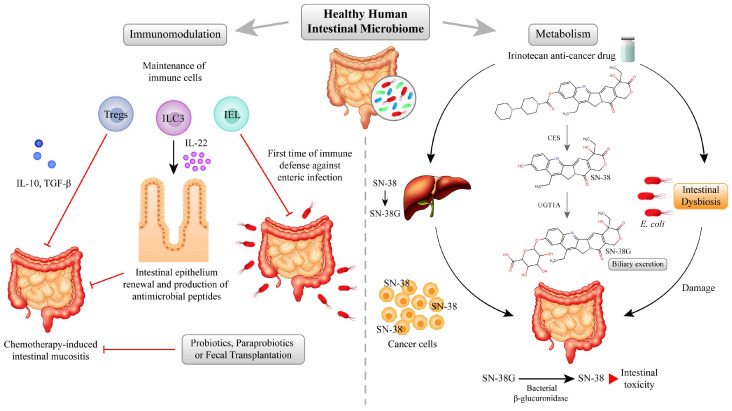
The interplay between microbiome and chemotherapy toxicity on the intestine. Changes in the human intestinal microbiome can influence the development of intestinal mucositis and the overall efficacy and side effects of chemotherapy. The two main mechanisms involved include (1) the modulation of the immune response and (2) alteration in the metabolism of the chemotherapy drug. The intestinal microbiome influences immune responses by interacting with gut-associated lymphoid tissue (GALT), critical for maintaining intestinal homeostasis. A healthy microbiota affects the immune system, favoring homeostasis. It maintains an anti-inflammatory microenvironment, rich in regulatory T cells (Tregs), to tolerate commensals. A healthy microbiota also generates antimicrobials to control pathogenic microorganisms by modulating the functions of intraepithelial lymphocytes (IEL), innate type 3 lymphoid cells (ILC3), and other immune cells within the mucosal layer. Tregs can prevent the development of intestinal mucositis by controlling inflammation. IL-22, produced by ILC3, assists in epithelial renewal and plays a crucial role in maintaining the integrity of the intestinal barrier, which helps control bacterial translocation and support IEL functions. Dysbiosis can affect intestinal immune homeostasis, increasing susceptibility to infections and inflammation, which can be restored with probiotics, paraprobiotics, or fecal transplantation. Fecal transplantation has shown potential to restore a balanced microbiome and mitigate chemotherapy-induced toxicity. Irinotecan is an anticancer drug metabolized in the liver to an active metabolite, SN-38. The conversion of irinotecan to SN-38 by carboxylesterase (CES) is crucial for its therapeutic efficacy. However, the subsequent metabolism of SN-38 by UDP glucuronosyltransferase family 1 member A1 (UGT1A1) can significantly impact its toxicity due to enterohepatic drug circulation. Changes in the intestinal microbiota, including the presence of specific bacterial species such as *Escherichia coli*, can favor the production of intestinal beta-glucuronidases and deconjugation of the irinotecan metabolite, SN-38G, resulting in increased levels of the active metabolite, SN-38, in the intestine and thereby enhancing toxicity. Chemical descriptors’ canonical SMILES were obtained from https://pubchem.ncbi.nlm.nih.gov/ (accessed on 28 May 2024) under the following PubChem identifiers: Irinotecan (60838); SN-38 (104842), and SN-38G (443154). Subsequently, 2D compound structures were generated with the Chemical Sketch Tool, available at https://www.rcsb.org/chemical-sketch (accessed on 28 May 2024) [115].

Figure 3 highlights the effects of the healthy human intestinal microbiome on immunomodulation and drug metabolism. It also emphasizes the significant influence of the gut microbiome on cancer chemotherapy’s toxicity, particularly gastrointestinal toxicity. This summary analysis reveals a complex, bidirectional relationship between microbiome composition and chemotherapy outcomes. The mechanisms involve the pathophysiological hallmarks of immunomodulation, inflammation, and drug metabolism.

## 5. Perspectives

The findings suggest that adapting the microbiome using diet, probiotics, or fecal microbiota transplantation (FMT) could effectively decrease gastrointestinal manifestations caused by chemotherapy. The variations in microbiome composition among individuals emphasize the importance of personalized cancer treatment. While incorporating microbiome profiling into clinical practice presents challenges, it can potentially tailor chemotherapy regimens, reduce toxicity, and enhance effectiveness. This approach could also help identify patients at a greater risk of severe side effects and adjust interventions accordingly.

Diet and lifestyle markedly affect the microbiome composition. Modulating these effects as non-pharmacological interventions would support cancer patients’ treatment. Increasing high-fiber diets and lowering processed food intake may help maintain a healthier microbiome, potentially reducing chemotherapy-related complications.

This study also emphasizes the importance of the microbiome in drug metabolism, especially in the activation and detoxification processes, underscoring the necessity of taking microbiome interactions into account in pharmacokinetic modeling and drug development.

The preclinical findings must be validated through more extensive clinical trials. Standardized guidelines for microbiome-based interventions should be established. Furthermore, investigating the microbiome’s impact on the effectiveness of other cancer treatments, like immunotherapy, could lead to new approaches for improving patient outcomes.

## 6. Conclusions

Interindividual variations in drug responsiveness, especially to anticancer drugs, have been troublesome in clinical settings. Considering the growing evidence regarding the intestinal microbiota’s roles in anticancer chemotherapy efficacy and intestinal side effects, an in-depth look at this topic should raise more attention to pursuing more effective and customized medicine. Identifying novel biomarkers of dysbiosis and microbiota-transferring interventions are needed, especially in many worldwide settings of poor sanitation and hygiene, where environmental-born contaminants may change the microbiota composition towards pathogenic dysbiosis and impaired microbiota maturity [31,116]. Such a condition may alter cancer treatment response and favor unwanted side effects, including intestinal mucositis. Notably, mucositis, per se, may aggravate the dysbiosis state, establishing a deleterious vicious cycle. Future research, including broader clinical trials and cohorts, is warranted to understand the mechanisms, shedding light on signaling pathways of treatment inadequacy under intestinal microbiota dysbiosis and translating these insights into clinical practice.

## Data Availability

The data presented in this study are available in the public Human Gut Microbiome Atlas databases, available at https://www.microbiomeatlas.org (accessed on 28 May 2024). The primary publications included the cohorts from the following bioprojects: Obesity (PRJEB4336); Crohn’s disease (PRJEB15371; PRJEB2054); Ulcerative colitis (PRJEB2054); Colon adenoma (PRJDB4176; PRJEB6070; PRJEB7774; PRJNA447983); Colorectal cancer (PRJDB4176; PRJEB10878; PRJEB12449; PRJEB27928; PRJEB6070; PRJEB7774; PRJNA447983; PRJNA531273); Non-small cell lung cancer (PRJEB22863); Renal Cancer (PRJEB22863).

## References

[1] Weng L., Zhang L., Peng Y., Huang R.S. (2013). Pharmacogenetics and Pharmacogenomics: A Bridge to Individualized Cancer Therapy. Pharmacogenomics.

[2] Doestzada M., Vila A.V., Zhernakova A., Koonen D.P.Y., Weersma R.K., Touw D.J., Kuipers F., Wijmenga C., Fu J. (2018). Pharmacomicrobiomics: A Novel Route towards Personalized Medicine?. Protein Cell.

[3] Yu Z.-K., Xie R.-L., You R., Liu Y.-P., Chen X.-Y., Chen M.-Y., Huang P.-Y. (2021). The Role of the Bacterial Microbiome in the Treatment of Cancer. BMC Cancer.

[4] Kundu P., Blacher E., Elinav E., Pettersson S. (2017). Our Gut Microbiome: The Evolving Inner Self. Cell.

[5] Bäckhed F., Fraser C.M., Ringel Y., Sanders M.E., Sartor R.B., Sherman P.M., Versalovic J., Young V., Finlay B.B. (2012). Defining a Healthy Human Gut Microbiome: Current Concepts, Future Directions, and Clinical Applications. Cell Host Microbe.

[6] David L.A., Maurice C.F., Carmody R.N., Gootenberg D.B., Button J.E., Wolfe B.E., Ling A.V., Devlin A.S., Varma Y., Fischbach M.A. (2014). Diet Rapidly and Reproducibly Alters the Human Gut Microbiome. Nature.

[7] Dethlefsen L., Huse S., Sogin M.L., Relman D.A. (2008). The Pervasive Effects of an Antibiotic on the Human Gut Microbiota, as Revealed by Deep 16S RRNA Sequencing. PLoS Biol..

[8] Sivan A., Corrales L., Hubert N., Williams J.B., Aquino-Michaels K., Earley Z.M., Benyamin F.W., Lei Y.M., Jabri B., Alegre M.-L. (2015). Commensal *Bifidobacterium* Promotes Antitumor Immunity and Facilitates Anti-PD-L1 Efficacy. Science.

[9] Freedberg D.E., Toussaint N.C., Chen S.P., Ratner A.J., Whittier S., Wang T.C., Wang H.H., Abrams J.A. (2015). Proton Pump Inhibitors Alter Specific Taxa in the Human Gastrointestinal Microbiome: A Crossover Trial. Gastroenterology.

[10] Forslund K., Hildebrand F., Nielsen T., Falony G., Le Chatelier E., Sunagawa S., Prifti E., Vieira-Silva S., Gudmundsdottir V., Pedersen H.K. (2015). Disentangling Type 2 Diabetes and Metformin Treatment Signatures in the Human Gut Microbiota. Nature.

[11] Sun L., Xie C., Wang G., Wu Y., Wu Q., Wang X., Liu J., Deng Y., Xia J., Chen B. (2018). Gut Microbiota and Intestinal FXR Mediate the Clinical Benefits of Metformin. Nat. Med..

[12] Zimmermann M., Zimmermann-Kogadeeva M., Wegmann R., Goodman A.L. (2019). Separating Host and Microbiome Contributions to Drug Pharmacokinetics and Toxicity. Science.

[13] Chau J., Yadav M., Liu B., Furqan M., Dai Q., Shahi S., Gupta A., Mercer K.N., Eastman E., Hejleh T.A. (2021). Prospective Correlation between the Patient Microbiome with Response to and Development of Immune-Mediated Adverse Effects to Immunotherapy in Lung Cancer. BMC Cancer.

[14] Weersma R.K., Zhernakova A., Fu J. (2020). Interaction between Drugs and the Gut Microbiome. Gut.

[15] Waring R.H., Harris R.M., Mitchell S.C. (2017). Drug Metabolism in the Elderly: A Multifactorial Problem?. Maturitas.

[16] Markle J.G.M., Frank D.N., Mortin-Toth S., Robertson C.E., Feazel L.M., Rolle-Kampczyk U., von Bergen M., McCoy K.D., Macpherson A.J., Danska J.S. (2013). Sex Differences in the Gut Microbiome Drive Hormone-Dependent Regulation of Autoimmunity. Science (1979).

[17] Allen J.M., Berg Miller M.E., Pence B.D., Whitlock K., Nehra V., Gaskins H.R., White B.A., Fryer J.D., Woods J.A. (2015). Voluntary and Forced Exercise Differentially Alters the Gut Microbiome in C57BL/6J Mice. J. Appl. Physiol..

[18] Montassier E., Gastinne T., Vangay P., Al-Ghalith G.A., Bruley des Varannes S., Massart S., Moreau P., Potel G., de La Cochetière M.F., Batard E. (2015). Chemotherapy-driven Dysbiosis in the Intestinal Microbiome. Aliment. Pharmacol. Ther..

[19] Ervin S.M., Ramanan S.V., Bhatt A.P. (2020). Relationship Between the Gut Microbiome and Systemic Chemotherapy. Dig. Dis. Sci..

[20] McQuade R.M., Stojanovska V., Abalo R., Bornstein J.C., Nurgali K. (2016). Chemotherapy-Induced Constipation and Diarrhea: Pathophysiology, Current and Emerging Treatments. Front. Pharmacol..

[21] Lalla R.V., Bowen J., Barasch A., Elting L., Epstein J., Keefe D.M., McGuire D.B., Migliorati C., Nicolatou-Galitis O., Peterson D.E. (2014). MASCC/ISOO Clinical Practice Guidelines for the Management of Mucositis Secondary to Cancer Therapy. Cancer.

[22] Wang Y., Sun L., Chen S., Guo S., Yue T., Hou Q., Feng M., Xu H., Liu Y., Wang P. (2019). The Administration of *Escherichia Coli Nissle 1917* Ameliorates Irinotecan-Induced Intestinal Barrier Dysfunction and Gut Microbial Dysbiosis in Mice. Life Sci..

[23] Lu D., Yan J., Liu F., Ding P., Chen B., Lu Y., Sun Z. (2019). Probiotics in Preventing and Treating Chemotherapy-Induced Diarrhea: A Meta-Analysis. Asia Pac. J. Clin. Nutr..

[24] Reyna-Figueroa J., Barrón-Calvillo E., García-Parra C., Galindo-Delgado P., Contreras-Ochoa C., Lagunas-Martínez A., Campos-Romero F.H., Silva-Estrada J.A., Limón-Rojas A.E. (2019). Probiotic Supplementation Decreases Chemotherapy-Induced Gastrointestinal Side Effects in Patients with Acute Leukemia. J. Pediatr. Hematol./Oncol..

[25] Bowen J.M., Gibson R.J., Coller J.K., Blijlevens N., Bossi P., Al-Dasooqi N., Bateman E.H., Chiang K., de Mooij C., Mayo B. (2019). Systematic Review of Agents for the Management of Cancer Treatment-Related Gastrointestinal Mucositis and Clinical Practice Guidelines. Support. Care Cancer.

[26] Elad S., Cheng K.K.F., Lalla R.V., Yarom N., Hong C., Logan R.M., Bowen J., Gibson R., Saunders D.P., Zadik Y. (2020). MASCC/ISOO Clinical Practice Guidelines for the Management of Mucositis Secondary to Cancer Therapy. Cancer.

[27] Robles Alonso V., Guarner F. (2013). Linking the Gut Microbiota to Human Health. Br. J. Nutr..

[28] Gomaa E.Z. (2020). Human Gut Microbiota/Microbiome in Health and Diseases: A Review. Antonie Van Leeuwenhoek.

[29] Rothschild D., Weissbrod O., Barkan E., Kurilshikov A., Korem T., Zeevi D., Costea P.I., Godneva A., Kalka I.N., Bar N. (2018). Environment Dominates over Host Genetics in Shaping Human Gut Microbiota. Nature.

[30] Levy M., Kolodziejczyk A.A., Thaiss C.A., Elinav E. (2017). Dysbiosis and the Immune System. Nat. Rev. Immunol..

[31] Leocádio P.C.L., Lopes S.C., Dias R.P., Alvarez-Leite J.I., Guerrant R.L., Malva J.O., Oriá R.B. (2021). The Transition From Undernutrition to Overnutrition Under Adverse Environments and Poverty: The Risk for Chronic Diseases. Front. Nutr..

[32] Simpson H.L., Campbell B.J. (2015). Review Article: Dietary Fibre-Microbiota Interactions. Aliment. Pharmacol. Ther..

[33] Nguyen N.K., Deehan E.C., Zhang Z., Jin M., Baskota N., Perez-Muñoz M.E., Cole J., Tuncil Y.E., Seethaler B., Wang T. (2020). Gut Microbiota Modulation with Long-Chain Corn Bran Arabinoxylan in Adults with Overweight and Obesity Is Linked to an Individualized Temporal Increase in Fecal Propionate. Microbiome.

[34] Chen T., Long W., Zhang C., Liu S., Zhao L., Hamaker B.R. (2017). Fiber-Utilizing Capacity Varies in Prevotella- versus Bacteroides-Dominated Gut Microbiota. Sci. Rep..

[35] Parada Venegas D., De la Fuente M.K., Landskron G., González M.J., Quera R., Dijkstra G., Harmsen H.J.M., Faber K.N., Hermoso M.A. (2019). Short Chain Fatty Acids (SCFAs)-Mediated Gut Epithelial and Immune Regulation and Its Relevance for Inflammatory Bowel Diseases. Front. Immunol..

[36] Chambers E.S., Byrne C.S., Morrison D.J., Murphy K.G., Preston T., Tedford C., Garcia-Perez I., Fountana S., Serrano-Contreras J.I., Holmes E. (2019). Dietary Supplementation with Inulin-Propionate Ester or Inulin Improves Insulin Sensitivity in Adults with Overweight and Obesity with Distinct Effects on the Gut Microbiota, Plasma Metabolome and Systemic Inflammatory Responses: A Randomised Cross-over Trial. Gut.

[37] Smith P.M., Howitt M.R., Panikov N., Michaud M., Gallini C.A., Bohlooly-y M., Glickman J.N., Garrett W.S. (2013). The Microbial Metabolites, Short-Chain Fatty Acids, Regulate Colonic Treg Cell Homeostasis. Science (1979).

[38] Haghikia A., Jörg S., Duscha A., Berg J., Manzel A., Waschbisch A., Hammer A., Lee D.-H., May C., Wilck N. (2015). Dietary Fatty Acids Directly Impact Central Nervous System Autoimmunity via the Small Intestine. Immunity.

[39] Lupp C., Robertson M.L., Wickham M.E., Sekirov I., Champion O.L., Gaynor E.C., Finlay B.B. (2007). Host-Mediated Inflammation Disrupts the Intestinal Microbiota and Promotes the Overgrowth of *Enterobacteriaceae*. Cell Host Microbe.

[40] Thim-Uam A., Makjaroen J., Issara-Amphorn J., Saisorn W., Wannigama D.L., Chancharoenthana W., Leelahavanichkul A. (2022). Enhanced Bacteremia in Dextran Sulfate-Induced Colitis in Splenectomy Mice Correlates with Gut Dysbiosis and LPS Tolerance. Int. J. Mol. Sci..

[41] Islam J., Koseki T., Watanabe K., Ardiansyah, Budijanto S., Oikawa A., Alauddin M., Goto T., Aso H., Komai M. (2017). Dietary Supplementation of Fermented Rice Bran Effectively Alleviates Dextran Sodium Sulfate-Induced Colitis in Mice. Nutrients.

[42] Vrieze A., Out C., Fuentes S., Jonker L., Reuling I., Kootte R.S., van Nood E., Holleman F., Knaapen M., Romijn J.A. (2014). Impact of Oral Vancomycin on Gut Microbiota, Bile Acid Metabolism, and Insulin Sensitivity. J. Hepatol..

[43] Palleja A., Mikkelsen K.H., Forslund S.K., Kashani A., Allin K.H., Nielsen T., Hansen T.H., Liang S., Feng Q., Zhang C. (2018). Recovery of Gut Microbiota of Healthy Adults Following Antibiotic Exposure. Nat. Microbiol..

[44] Bartlett J.G. (2002). Clinical Practice. Antibiotic-Associated Diarrhea. N. Engl. J. Med..

[45] Willing B.P., Russell S.L., Finlay B.B. (2011). Shifting the Balance: Antibiotic Effects on Host-Microbiota Mutualism. Nat. Rev. Microbiol..

[46] Sharma R., Young C., Neu J. (2010). Molecular Modulation of Intestinal Epithelial Barrier: Contribution of Microbiota. BioMed Res. Int..

[47] Feng Y., Huang Y., Wang Y., Wang P., Song H., Wang F. (2019). Antibiotics Induced Intestinal Tight Junction Barrier Dysfunction Is Associated with Microbiota Dysbiosis, Activated NLRP3 Inflammasome and Autophagy. PLoS ONE.

[48] von Itzstein M.S., Gonugunta A.S., Sheffield T., Homsi J., Dowell J.E., Koh A.Y., Raj P., Fattah F., Wang Y., Basava V.S. (2022). Association between Antibiotic Exposure and Systemic Immune Parameters in Cancer Patients Receiving Checkpoint Inhibitor Therapy. Cancers.

[49] Chi L., Bian X., Gao B., Tu P., Ru H., Lu K. (2017). The Effects of an Environmentally Relevant Level of Arsenic on the Gut Microbiome and Its Functional Metagenome. Toxicol. Sci..

[50] Chi L., Tu P., Ru H., Lu K. (2021). Studies of Xenobiotic-Induced Gut Microbiota Dysbiosis: From Correlation to Mechanisms. Gut Microbes.

[51] Zhao Y., Zhou C., Wu C., Guo X., Hu G., Wu Q., Xu Z., Li G., Cao H., Li L. (2020). Subchronic Oral Mercury Caused Intestinal Injury and Changed Gut Microbiota in Mice. Sci. Total Environ..

[52] Pinto D.V., Raposo R.S., Matos G.A., Alvarez-Leite J.I., Malva J.O., Oriá R.B. (2020). Methylmercury Interactions with Gut Microbiota and Potential Modulation of Neurogenic Niches in the Brain. Front. Neurosci..

[53] Kittle R.P., McDermid K.J., Muehlstein L., Balazs G.H. (2018). Effects of Glyphosate Herbicide on the Gastrointestinal Microflora of Hawaiian Green Turtles (*Chelonia mydas*) Linnaeus. Mar. Pollut. Bull..

[54] Joly C., Gay-Quéheillard J., Léké A., Chardon K., Delanaud S., Bach V., Khorsi-Cauet H. (2013). Impact of Chronic Exposure to Low Doses of Chlorpyrifos on the Intestinal Microbiota in the Simulator of the Human Intestinal Microbial Ecosystem (SHIME) and in the Rat. Environ. Sci. Pollut. Res. Int..

[55] Nasuti C., Coman M.M., Olek R.A., Fiorini D., Verdenelli M.C., Cecchini C., Silvi S., Fedeli D., Gabbianelli R. (2016). Changes on Fecal Microbiota in Rats Exposed to Permethrin during Postnatal Development. Environ. Sci. Pollut. Res. Int..

[56] Montassier E., Batard E., Massart S., Gastinne T., Carton T., Caillon J., Le Fresne S., Caroff N., Hardouin J.B., Moreau P. (2014). 16S RRNA Gene Pyrosequencing Reveals Shift in Patient Faecal Microbiota during High-Dose Chemotherapy as Conditioning Regimen for Bone Marrow Transplantation. Microb. Ecol..

[57] Motoori M., Yano M., Miyata H., Sugimura K., Saito T., Omori T., Fujiwara Y., Miyoshi N., Akita H., Gotoh K. (2017). Randomized Study of the Effect of Synbiotics during Neoadjuvant Chemotherapy on Adverse Events in Esophageal Cancer Patients. Clin. Nutr..

[58] Huang Y., Yang W., Liu H., Duan J., Zhang Y., Liu M., Li H., Hou Z., Wu K.K. (2012). Effect of High-Dose Methotrexate Chemotherapy on Intestinal *Bifidobacteria*, *Lactobacillus* and *Escherichia Coli* in Children with Acute Lymphoblastic Leukemia. Exp. Biol. Med..

[59] Rajagopala S.V., Singh H., Yu Y., Zabokrtsky K.B., Torralba M.G., Moncera K.J., Frank B., Pieper R., Sender L., Nelson K.E. (2020). Persistent Gut Microbial Dysbiosis in Children with Acute Lymphoblastic Leukemia (ALL) During Chemotherapy. Microb. Ecol..

[60] Lin X.B., Dieleman L.A., Ketabi A., Bibova I., Sawyer M.B., Xue H., Field C.J., Baracos V.E., Gänzle M.G. (2012). Irinotecan (CPT-11) Chemotherapy Alters Intestinal Microbiota in Tumour Bearing Rats. PLoS ONE.

[61] Stringer A.M., Gibson R.J., Logan R.M., Bowen J.M., Yeoh A.S.J., Keefe D.M.K. (2008). Faecal Microflora and Beta-Glucuronidase Expression Are Altered in an Irinotecan-Induced Diarrhea Model in Rats. Cancer Biol. Ther..

[62] Zeng M.Y., Inohara N., Nuñez G. (2017). Mechanisms of Inflammation-Driven Bacterial Dysbiosis in the Gut. Mucosal Immunol..

[63] Wu C.-H., Ko J.-L., Liao J.-M., Huang S.-S., Lin M.-Y., Lee L.-H., Chang L.-Y., Ou C.-C. (2019). D-Methionine Alleviates Cisplatin-Induced Mucositis by Restoring the Gut Microbiota Structure and Improving Intestinal Inflammation. Ther. Adv. Med. Oncol..

[64] Klaassen C.D., Cui J.Y. (2015). Review: Mechanisms of How the Intestinal Microbiota Alters the Effects of Drugs and Bile Acids. Drug Metab. Dispos..

[65] Jaye K., Li C.G., Bhuyan D.J. (2021). The Complex Interplay of Gut Microbiota with the Five Most Common Cancer Types: From Carcinogenesis to Therapeutics to Prognoses. Crit. Rev. Oncol. Hematol..

[66] Kaźmierczak-Siedlecka K., Daca A., Fic M., van de Wetering T., Folwarski M., Makarewicz W. (2020). Therapeutic Methods of Gut Microbiota Modification in Colorectal Cancer Management—Fecal Microbiota Transplantation, Prebiotics, Probiotics, and Synbiotics. Gut Microbes.

[67] Ribeiro R.A., Wanderley C.W.S., Wong D.V.T., Mota J.M.S.C., Leite C.A.V.G., Souza M.H.L.P., Cunha F.Q., Lima-Júnior R.C.P. (2016). Irinotecan- and 5-Fluorouracil-Induced Intestinal Mucositis: Insights into Pathogenesis and Therapeutic Perspectives. Cancer Chemother. Pharmacol..

[68] Sougiannis A.T., VanderVeen B.N., Davis J.M., Fan D., Murphy E.A. (2021). Understanding Chemotherapy-Induced Intestinal Mucositis and Strategies to Improve Gut Resilience. Am. J. Physiol. Gastrointest. Liver Physiol..

[69] Dranitsaris G., Maroun J., Shah A. (2005). Severe Chemotherapy-Induced Diarrhea in Patients with Colorectal Cancer: A Cost of Illness Analysis. Support Care Cancer.

[70] Mayo B.J., Stringer A.M., Bowen J.M., Bateman E.H., Keefe D.M. (2017). Irinotecan-Induced Mucositis: The Interactions and Potential Role of GLP-2 Analogues. Cancer Chemother. Pharmacol..

[71] Bailly C. (2019). Irinotecan: 25 Years of Cancer Treatment. Pharmacol. Res..

[72] Wong D.V.T., Lima-Júnior R.C.P., Carvalho C.B.M., Borges V.F., Wanderley C.W.S., Bem A.X.C., Leite C.A.V.G., Teixeira M.A., Batista G.L.P., Silva R.L. (2015). The Adaptor Protein Myd88 Is a Key Signaling Molecule in the Pathogenesis of Irinotecan-Induced Intestinal Mucositis. PLoS ONE.

[73] Wong D.V.T., Holanda R.B.F., Cajado A.G., Bandeira A.M., Pereira J.F.B., Amorim J.O., Torres C.S., Ferreira L.M.M., Lopes M.H.S., Oliveira R.T.G. (2021). TLR4 Deficiency Upregulates TLR9 Expression and Enhances Irinotecan-Related Intestinal Mucositis and Late-Onset Diarrhoea. Br. J. Pharmacol..

[74] van Vliet M.J., Harmsen H.J.M., de Bont E.S.J.M., Tissing W.J.E. (2010). The Role of Intestinal Microbiota in the Development and Severity of Chemotherapy-Induced Mucositis. PLoS Pathog..

[75] Newton K., Dixit V.M. (2012). Signaling in Innate Immunity and Inflammation. Cold Spring Harb. Perspect. Biol..

[76] Lee C.S., Ryan E.J., Doherty G.A. (2014). Gastro-Intestinal Toxicity of Chemotherapeutics in Colorectal Cancer: The Role of Inflammation. World J. Gastroenterol..

[77] Lima-Júnior R.C.P., Freitas H.C., Wong D.V.T., Wanderley C.W.S., Nunes L.G., Leite L.L., Miranda S.P., Souza M.H.L.P., Brito G.A.C., Magalhães P.J.C. (2014). Targeted Inhibition of IL-18 Attenuates Irinotecan-Induced Intestinal Mucositis in Mice. Br. J. Pharmacol..

[78] Arifa R.D.N., Madeira M.F.M., De Paula T.P., Lima R.L., Tavares L.D., Menezes-garcia Z. (2014). Inflammasome Activation Is Reactive Oxygen Species Dependent and Mediates Irinotecan-Induced Mucositis through IL-1 b and IL-18 in Mice. Am. J. Pathol..

[79] Wong D.V.T., Ribeiro-Filho H.V., Wanderley C.W.S., Leite C.A.V.G., Lima J.B., Assef A.N.B., Cajado A.G., Batista G.L.P., González R.H., Silva K.O. (2019). SN-38, the Active Metabolite of Irinotecan, Inhibits the Acute Inflammatory Response by Targeting Toll-like Receptor 4. Cancer Chemother. Pharmacol..

[80] Secombe K.R., Crame E.E., Tam J.S.Y., Wardill H.R., Gibson R.J., Coller J.K., Bowen J.M. (2022). Intestinal Toll-like Receptor 4 Knockout Alters the Functional Capacity of the Gut Microbiome Following Irinotecan Treatment. Cancer Chemother. Pharmacol..

[81] Liu Y., Yang M., Tang L., Wang F., Huang S., Liu S., Lei Y., Wang S., Xie Z., Wang W. (2022). TLR4 Regulates RORγt(+) Regulatory T-Cell Responses and Susceptibility to Colon Inflammation through Interaction with *Akkermansia muciniphila*. Microbiome.

[82] Fernandes C., Wanderley C.W.S., Silva C.M.S., Muniz H.A., Teixeira M.A., Souza N.R.P., Cândido A.G.F., Falcão R.B., Souza M.H.L.P., Almeida P.R.C. (2018). Role of Regulatory T Cells in Irinotecan-Induced Intestinal Mucositis. Eur. J. Pharm. Sci..

[83] Ohnmacht C., Park J.-H., Cording S., Wing J.B., Atarashi K., Obata Y., Gaboriau-Routhiau V., Marques R., Dulauroy S., Fedoseeva M. (2015). MUCOSAL IMMUNOLOGY. The Microbiota Regulates Type 2 Immunity through RORγt^+^ T Cells. Science.

[84] Cervantes-Barragan L., Chai J.N., Tianero M.D., Di Luccia B., Ahern P.P., Merriman J., Cortez V.S., Caparon M.G., Donia M.S., Gilfillan S. (2017). *Lactobacillus reuteri* Induces Gut Intraepithelial CD4(+)CD8αα(+) T Cells. Science.

[85] Hepworth M.R., Monticelli L.A., Fung T.C., Ziegler C.G.K., Grunberg S., Sinha R., Mantegazza A.R., Ma H.-L., Crawford A., Angelosanto J.M. (2013). Innate Lymphoid Cells Regulate CD4+ T-Cell Responses to Intestinal Commensal Bacteria. Nature.

[86] Cerf-Bensussan N., Gaboriau-Routhiau V. (2010). The Immune System and the Gut Microbiota: Friends or Foes?. Nat. Rev. Immunol..

[87] Mazmanian S.K., Round J.L., Kasper D.L. (2008). A Microbial Symbiosis Factor Prevents Intestinal Inflammatory Disease. Nature.

[88] Dalile B., Van Oudenhove L., Vervliet B., Verbeke K. (2019). The Role of Short-Chain Fatty Acids in Microbiota-Gut-Brain Communication. Nat. Rev. Gastroenterol. Hepatol..

[89] Sun M., Wu W., Liu Z., Cong Y. (2017). Microbiota Metabolite Short Chain Fatty Acids, GPCR, and Inflammatory Bowel Diseases. J. Gastroenterol..

[90] Visekruna A., Luu M. (2021). The Role of Short-Chain Fatty Acids and Bile Acids in Intestinal and Liver Function, Inflammation, and Carcinogenesis. Front. Cell Dev. Biol..

[91] Zhang Z., Zhang H., Chen T., Shi L., Wang D., Tang D. (2022). Regulatory Role of Short-Chain Fatty Acids in Inflammatory Bowel Disease. Cell Commun. Signal..

[92] Venkatraman A., Ramakrishna B.S., Pulimood A.B. (1999). Butyrate Hastens Restoration of Barrier Function after Thermal and Detergent Injury to Rat Distal Colon in Vitro. Scand. J. Gastroenterol..

[93] Kelly D., Campbell J.I., King T.P., Grant G., Jansson E.A., Coutts A.G.P., Pettersson S., Conway S. (2004). Commensal Anaerobic Gut Bacteria Attenuate Inflammation by Regulating Nuclear-Cytoplasmic Shuttling of PPAR-Gamma and RelA. Nat. Immunol..

[94] Pandey S., Kawai T., Akira S. (2014). Microbial Sensing by Toll-like Receptors and Intracellular Nucleic Acid Sensors. Cold Spring Harb. Perspect. Biol..

[95] Wei L., Wen X.-S., Xian C.J. (2021). Chemotherapy-Induced Intestinal Microbiota Dysbiosis Impairs Mucosal Homeostasis by Modulating Toll-like Receptor Signaling Pathways. Int. J. Mol. Sci..

[96] Buela K.-A.G., Omenetti S., Pizarro T.T. (2015). Cross-Talk between Type 3 Innate Lymphoid Cells and the Gut Microbiota in Inflammatory Bowel Disease. Curr. Opin. Gastroenterol..

[97] Crellin N.K., Trifari S., Kaplan C.D., Satoh-Takayama N., Di Santo J.P., Spits H. (2010). Regulation of Cytokine Secretion in Human CD127(+) LTi-like Innate Lymphoid Cells by Toll-like Receptor 2. Immunity.

[98] Wang Y., Mumm J.B., Herbst R., Kolbeck R., Wang Y. (2017). IL-22 Increases Permeability of Intestinal Epithelial Tight Junctions by Enhancing Claudin-2 Expression. J. Immunol..

[99] Yeung C.-Y., Chan W.-T., Jiang C.-B., Cheng M.-L., Liu C.-Y., Chang S.-W., Chiang Chiau J.-S., Lee H.-C. (2015). Amelioration of Chemotherapy-Induced Intestinal Mucositis by Orally Administered Probiotics in a Mouse Model. PLoS ONE.

[100] Huang L., Chiang Chiau J.-S., Cheng M.-L., Chan W.-T., Jiang C.-B., Chang S.-W., Yeung C.-Y., Lee H.-C. (2019). SCID/NOD Mice Model for 5-FU Induced Intestinal Mucositis: Safety and Effects of Probiotics as Therapy. Pediatr. Neonatol..

[101] Quintanilha M.F., Miranda V.C., Souza R.O., Gallotti B., Cruz C., Santos E.A., Alvarez-Leite J.I., Jesus L.C.L., Azevedo V., Trindade L.M. (2022). *Bifidobacterium Longum* Subsp. Longum 5(1A) Attenuates Intestinal Injury against Irinotecan-Induced Mucositis in Mice. Life Sci..

[102] Chang C.-W., Lee H.-C., Li L.-H., Chiang Chiau J.-S., Wang T.-E., Chuang W.-H., Chen M.-J., Wang H.-Y., Shih S.-C., Liu C.-Y. (2020). Fecal Microbiota Transplantation Prevents Intestinal Injury, Upregulation of Toll-Like Receptors, and 5-Fluorouracil/Oxaliplatin-Induced Toxicity in Colorectal Cancer. Int. J. Mol. Sci..

[103] Wardill H.R., van der Aa S.A.R., da Silva Ferreira A.R., Havinga R., Tissing W.J.E., Harmsen H.J.M. (2021). Antibiotic-Induced Disruption of the Microbiome Exacerbates Chemotherapy-Induced Diarrhoea and Can Be Mitigated with Autologous Faecal Microbiota Transplantation. Eur. J. Cancer.

[104] Nobre L.M.S., da Silva Lopes M.H., Geraix J., Cajado A.G., Silva J.M.R., Ribeiro L.R., Freire R.S., Cavalcante D.I.M., Wong D.V.T., Alves A.P.N.N. (2022). Paraprobiotic *Enterococcus faecalis* EC-12 Prevents the Development of Irinotecan-Induced Intestinal Mucositis in Mice. Life Sci..

[105] Nobre L.M.S., Fernandes C., Florêncio K.G.D., Alencar N.M.N., Wong D.V.T., Lima-Júnior R.C.P. (2023). Could Paraprobiotics Be a Safer Alternative to Probiotics for Managing Cancer Chemotherapy-Induced Gastrointestinal Toxicities?. Braz. J. Med. Biol. Res..

[106] Mego M., Chovanec J., Vochyanova-Andrezalova I., Konkolovsky P., Mikulova M., Reckova M., Miskovska V., Bystricky B., Beniak J., Medvecova L. (2015). Prevention of Irinotecan Induced Diarrhea by Probiotics: A Randomized Double Blind, Placebo Controlled Pilot Study. Complement. Ther. Med..

[107] Li H., He J., Jia W. (2016). The Influence of Gut Microbiota on Drug Metabolism and Toxicity. Expert Opin. Drug Metab. Toxicol..

[108] Chamseddine A.N., Ducreux M., Armand J.-P., Paoletti X., Satar T., Paci A., Mir O. (2019). Intestinal Bacterial β-Glucuronidase as a Possible Predictive Biomarker of Irinotecan-Induced Diarrhea Severity. Pharmacol. Ther..

[109] Takasuna K., Hagiwara T., Hirohashi M., Kato M., Nomura M., Nagai E., Yokoi T., Kamataki T. (1996). Involvement of Beta-Glucuronidase in Intestinal Microflora in the Intestinal Toxicity of the Antitumor Camptothecin Derivative Irinotecan Hydrochloride (CPT-11) in Rats. Cancer Res..

[110] Kehrer D.F., Sparreboom A., Verweij J., de Bruijn P., Nierop C.A., van de Schraaf J., Ruijgrok E.J., de Jonge M.J. (2001). Modulation of Irinotecan-Induced Diarrhea by Cotreatment with Neomycin in Cancer Patients. Clin. Cancer Res..

[111] Pedroso S.H.S.P., Vieira A.T., Bastos R.W., Oliveira J.S., Cartelle C.T., Arantes R.M.E., Soares P.M.G., Generoso S.V., Cardoso V.N., Teixeira M.M. (2015). Evaluation of Mucositis Induced by Irinotecan after Microbial Colonization in Germ-Free Mice. Microbiology.

[112] Vermes A., Kuijper E.J., Guchelaar H.-J., Dankert J. (2003). An in Vitro Study on the Active Conversion of Flucytosine to Fluorouracil by Microorganisms in the Human Intestinal Microflora. Chemotherapy.

[113] Alexander J.L., Wilson I.D., Teare J., Marchesi J.R., Nicholson J.K., Kinross J.M. (2017). Gut Microbiota Modulation of Chemotherapy Efficacy and Toxicity. Nat. Rev. Gastroenterol. Hepatol..

[114] Viaud S., Saccheri F., Mignot G., Yamazaki T., Daillère R., Hannani D., Enot D.P., Pfirschke C., Engblom C., Pittet M.J. (2013). The Intestinal Microbiota Modulates the Anticancer Immune Effects of Cyclophosphamide. Science (1979).

[115] Berman H.M., Westbrook J., Feng Z., Gilliland G., Bhat T.N., Weissig H., Shindyalov I.N., Bourne P.E. (2000). The Protein Data Bank. Nucleic Acids Res..

[116] Oriá R.B., Empadinhas N., Malva J.O. (2020). Editorial: Interplay Between Nutrition, the Intestinal Microbiota and the Immune System. Front. Immunol..

